# Current Immunotherapeutic Approaches in T Cell Non-Hodgkin Lymphomas

**DOI:** 10.3390/cancers10090339

**Published:** 2018-09-18

**Authors:** Teresa Poggio, Justus Duyster, Anna L. Illert

**Affiliations:** 1 Department of Hematology and Oncology, Medical Center, Faculty of Medicine, University of Freiburg, 79106 Freiburg, Germany; teresa.poggio@uniklinik-freiburg.de (T.P.); justus.duyster@uniklinik-freiburg.de (J.D.); 2 Faculty of Biology, Albert Ludwigs University of Freiburg, 79104 Freiburg, Germany; 3 German Cancer Consortium (DKTK) Partner Site Freiburg, 79106 Freiburg, Germany; 4 German Cancer Research Center (DKFZ), 69120 Heidelberg, Germany

**Keywords:** brentuximab vedotin, chimeric antigen receptor (CAR)-T cell, checkpoint inhibitors, monoclonal antibodies, T cell non-Hodgkin lymphoma (T-NHL)

## Abstract

T cell non-Hodgkin lymphoma (T-NHL) is a rare and heterogeneous group of neoplasms of the lymphoid system. With the exception of a few relatively indolent entities, T-NHL is typically aggressive, treatment resistant, and associated with poor prognosis. Relatively few options with proven clinical benefit are available for patients with relapsed or refractory disease. Immunotherapy has emerged as a promising treatment for the management of patients with hematological malignancies. The identification of tumor antigens has provided a large number of potential targets. Therefore, several monoclonal antibodies (alemtuzumab, SGN-30, brentuximab vedotin, and mogamulizumab), directed against tumor antigens, have been investigated in different subtypes of T-NHL. In addition to targeting antigens involved in cancer cell physiology, antibodies can stimulate immune effector functions or counteract immunosuppressive mechanisms. Chimeric antigen receptor (CAR)-T cells directed against CD30 and immune checkpoint inhibitors are currently being investigated in clinical trials. In this review, we summarize the currently available clinical evidence for immunotherapy in T-NHL, focusing on the results of clinical trials using first generation monoclonal antibodies, new immunotherapeutic agents, immune checkpoint inhibitors, and CAR-T cell therapies.

## 1. Introduction

Non-Hodgkin lymphoma (NHL) encompasses a heterogeneous group of malignant neoplasms of the lymphoid system accounting for about 4% of all cancers in the United States. For 2018, the American Cancer Society estimates that about 74,680 people will be diagnosed with NHL, and around 19,910 people will die from this disease [1]. NHL malignancies arise from clonal expansion of B-, T-, or natural killer (NK) cells. B-NHL occurs more frequent than T cell origin subtypes, which account for 10–15% of all NHL. 

The 2016 World Health Organizations updated the classification of lymphoid neoplasms to include 26 mature T cell neoplasms [2]. Among these, the most common subgroup is peripheral T cell lymphoma-not otherwise specified (PTCL-NOS; 26%), followed by angioimmunoblastic lymphoma (18%). Anaplastic large-cell lymphoma (ALCL) accounts for 12% of T-NHL cases, of which 6.5% are anaplastic lymphoma kinase (ALK) positive and 5.5% are ALK negative. Natural killer/T cell lymphoma (NKTCL) and adult T cell leukemia/lymphoma (ATL) represent 12% and 10% of cases, respectively [3]. Among the mature T cell neoplasms, the primary cutaneous lymphomas represent a heterogeneous group of extranodal NHL confined to the skin. Approximately 71% of these are cutaneous T cell lymphomas (CTCL) and they comprise mycosis fungoides (MF), Sézary syndrome (SS), and cutaneous CD30+ lymphoproliferative disorder [4,5]. The incidence of T-NHL is higher in men and the median age at diagnosis is 62 years. However, the median age differs among subtypes. For instance, ALK^pos^ ALCL primarily affects children and young adults with a median age of 33 years [6].

Standard first-line treatment for NHL malignancies includes anthracycline-based chemotherapy, such as cyclophosphamide, doxorubicin, vincristine, and prednisone (CHOP) or CHOP-like regimens. Traditionally, T-NHL has been treated according to chemotherapy schedules established for aggressive B cell lymphomas. However, the five-year overall survival (OS) and the event-free survival (EFS) rates for PTCL patients are significantly lower compared to B cell lymphoma patients, with an OS of 41% versus 53% and EFS of 33% versus 42%, respectively. Specifically, the complete response (CR) rates associated with standard treatment in NKTCL, AITL, ATL, and ALCL patients are 58%, 42%, 25%, and 66%, respectively [7,8]. The reported five-year OS in patients with ALCL is higher (56%) compared to NKTCL (42%), AITL (32%), and ATL (14%) patients [3]. Almost one-third of patients with PTCL experience progressive disease during primary therapy, and the median OS after relapse is 5.5 months. In a relapsed setting, the median OS increased to 6.5 months in patients that received chemotherapy, with a median OS in PTCL-NOS, AITL, and ALCL patients of 6.5 months, 7.7 months, and 3 months, respectively [9].

While exhibiting cytotoxic effects, most chemotherapeutic agents, due to their lack of specificity, negatively affect different types of normal cells as well, leading to adverse side effects in multiple organ systems [10]. The most common non-hematological short-term adverse effects of chemotherapy include fatigue, alopecia, nausea, vomiting, malaise, diarrhea, mucositis, and rashes [11]. The most frequent hematological adverse events (AEs) of chemotherapeutic agents are leukocytopenia, neutropenia, anemia, and thrombocytopenia, resulting in increased susceptibility to infections and an elevated risk of bleeding [12,13]. In recent years, several studies have reported an elevated risk of secondary malignancies after preceding curative therapy for aggressive NHL as well as late non-neoplastic events. Most studies reported a higher incidence of myelodysplastic syndrome/acute myeloid leukemia (MDS/AML); several solid tumors, including cancers of the bladder, lungs, gastrointestinal (GI) tract, head and neck, thyroid, and central nervous system (CNS); and sarcoma, breast cancer, and mesothelioma [14,15,16,17,18,19,20]. In a retrospective study conducted by the European Organization for Research and Treatment of Cancer (EORTC), late non-neoplastic events were observed in 46% of 757 patients consistently treated with doxorubicin-based chemotherapy since 1980 (median follow-up of 9.4 years). The most common late complications were cardiac disease and female infertility, and the 15-year cumulative incidence rates were 20% and 29%, respectively. Other late events included male infertility, disabling neuropathy, renal insufficiency, gastrointestinal (GI) toxicity, and lung fibrosis [21].

The generally poor outcome observed in T-NHL patients, due to unresponsiveness to standard chemotherapy, relapses after treatment, and toxicity-related events, highlights the urgent need for alternative treatment strategies. 

NHL malignancies occur in immune-rich lymphoid tissues, expressing co-stimulatory molecules as well as unique tumor antigens, which render them attractive targets for immunotherapy. This therapeutic approach stems from immunosurveillance theory, which describes the ability of the immune system to detect and prevent tumor development. Immunosurveillance is carried out by the humoral and cellular components of the innate and adaptive immune system. The process of T cell activation during the adaptive immune response requires two signals delivered by antigen-presenting cells (APCs). The first signal is the antigen presentation by the major histocompatibility complex (MHC) molecules on APCs to the corresponding T cell receptor (TCR) on naive T cells. The second signal is the co-stimulatory signal provided by molecules on APCs, which engage with specific co-stimulatory receptors on T cells. The best-characterized T cell co-stimulatory pathway involves the CD28 receptor on T cells, which binds to two co-stimulatory molecules, B7-1 (CD80) and B7-2 (CD86), on APCs. The process of T cell activation is tightly regulated by the balance of co-stimulatory and co-inhibitory signals, which maintain self-tolerance and prevent autoimmunity [22]. One mechanism through which tumors escape immune surveillance is by overexpressing immunosuppressive surface ligands that interact with T cell molecules, such as cytotoxic T-lymphocyte–associated antigen 4 (CTLA-4) or programmed cell death protein (PD-1), leading to a dysfunctional T cells state known as T cell exhaustion. These recent discoveries led to the development of immune checkpoint inhibitors—specific antibodies that antagonize the immunosuppressive interactions between the tumor cell and T cells [23]. In recent years, several clinical trials have confirmed the efficacy of immune checkpoint inhibitors blocking the CTLA-4 and PD-1 pathways in different cancer entities, thus enabling approval of these immunotherapies for the treatment of melanoma, renal cell carcinoma, and non-small cell lung cancer.

In classical Hodgkin Lymphoma (cHL) genomic amplification of the chromosomal locus 9p24.1, containing the genes encoding the inhibitory immune-checkpoint proteins programmed cell death ligand-1 (PD-L1) and PD-L2, which results in increased PD-L1 expression by Hodgkin Reed Stenberg (HRS) cells, have been reported. Accordingly, a very high response rate to PD-1 blockade has been observed in relapsed/refractory (R/R) cHL using nivolumab or pembrolixumab, with high overall response rates (ORR) (65–87%) and complete response (CR) rates (16–17%) [24,25].

Based on these results, in 2016, the U.S. Food and Drug Administration (FDA) granted accelerated approval to nivolumab for the treatment of patients with cHL. 

Another emerging approach to boost and activate T cell response against tumor cells is chimeric antigen receptor (CAR)-T cell immunotherapy. CAR-T cells are autologous T-lymphocytes genetically modified to express CAR constructs targeting a specific antigen on the tumor cells. The constructs are composed of a single chain variable (scFv) of a monoclonal antibody joined to the intracellular T cell signaling domain of the TCR CD3-ζ chain. Co-stimulatory molecules, such as CD28 and 4-1 BB, have been engineered to the signal transduction region in second and third generation CARs [26]. CAR-T cell therapies allow for the redirection and activation of effector T cells toward a specific tumor-associated antigen and are independent of major histocompatibility complex (MHC) restriction.

Data from clinical trials using anti-CD19 CAR-T cells have produced strong results in the treatment of B cell malignancies [27,28]. Several recently published studies showed that treatment of pediatric patients with R/R acute lymphoblastic leukemia achieved a complete response (CR) rate of 70–90% after infusions of T cells transduced with an anti-CD19 CAR [29,30].

In 2017, the FDA granted breakthrough designation to two different CAR-T cell therapies, CTL019 (tisagenlecleucel) for the treatment of R/R pediatric and young adult patients with B cell acute lymphoblastic leukemia (ALL), as well as Yescarta (axicabtagene ciloleucel). Yescarta has been approved for the treatment of adult patients with R/R large B cell lymphoma after two or more lines of systemic therapy, including diffuse large B cell lymphoma (DLBCL) not otherwise specified, primary mediastinal large B cell lymphoma (PMBCL), high grade B cell lymphoma, and DLBCL arising from follicular lymphoma. 

The promising results of checkpoint inhibitors and CAR-T cell therapies in HL and B cell lymphoma are prompting an increased number of clinical trials aimed at evaluating the effects in specific NHL subtypes. In this review, we aim to summarize the current clinical data on immunotherapy for relapsed and/or refractory T-NHL. Specifically, we provide an overview of the first generation monoclonal antibodies, new immunotherapeutic agents, checkpoint inhibitors, and CAR-T cells, and we discuss the available clinical evidence of these agents in T-NHL.

## 2. Monoclonal Antibodies

### 2.1. Alemtuzumab (Anti-CD52 Monoclonal Antibody)

Alemtuzumab is a humanized IgG1 kappa monoclonal antibody directed against the CD52 antigen, which is mostly expressed by B- and T-lymphocytes. Clinical activity of alemtuzumab as a monotherapy has been evaluated in mycoses fungoides (MF), Sézary syndrome (SS), and R/R peripheral T cell lymphoma (PTCL) [31,32]. The ORR in SS and MF patients was 55%, with CR in 32% of patients (Table 1) [31]. Treatment with alemtuzumab was associated with acceptable hematological toxicities and infection complications. Overall, 18% of patients experienced grade 4 neutropenia and cytomegalovirus (CMV) reactivation was reported in four (18%) patients.

In patients with PTCL, the ORR was 36%, with 21% achieving CR [32]. Pancytopenia and cytomegalovirus (CMV) reactivation were the most commonly reported adverse AEs. Another study showed long-lasting remission in SS, with an ORR of 70%, but limited efficacy in MF, with an ORR of 25% [33]. A grade 3 or higher infectious AE was reported in 62% of patients and 26% of patients experienced hematological toxicity. In a phase II study of alemtuzumab in 10 patients with PTCL and MF, an ORR of 60% (two complete responses and four partial responses) was reported [34]. Alemtuzumab has been combined with different forms and schedules of CHOP for the treatment of R/R [35] or newly diagnosed PTCL patients [36,37,38]. The combination of CHOP-14 [38], CHOP-21 [36], and CHOP-28 [37] with alemtuzumab showed high CR rates (60–71%), but was associated with high relapse rates and infection-related AEs. Among these studies, neutropenia was the most frequent hematologic toxicity. Due to heterogeneous patient populations, different doses of alemtuzumab, and variable chemotherapy protocols, comparison of the different phase II studies is intricate. 

In November 2014, the FDA approved Lemtrada (alemtuzumab) for the treatment of patients with the relapsing form of multiple sclerosis (MS).

### 2.2. Brentuximab Vedotin (Antibody-Drug Conjugate Directed against CD30)

CD30 (Ki-1) is highly expressed in malignant lymphoid cells, including B and T cell leukemia cells, Reed-Sternberg cells of Hodgkin lymphoma, and some NHL, both at diagnosis and relapse of disease [52,53,54,55,56]. Therefore, CD30 represents an attractive and validated target for immunotherapy in T-NHL. Monoclonal antibodies that target CD30, like SGN-30, showed an acceptable safety profile but only modest clinical activity, which has been observed in patients with primary cutaneous ALCL (43% CR rate) and in ALCL patients (17% ORR) (Table 1) [39,40]. 

To enhance the antitumor activity of SGN-30, the microtubule-disrupting agent mono-methylauristatin E (MMAE) was linked to the anti-CD30 monoclonal antibody, producing the anti-CD30 antibody-drug conjugate brentuximab vedotin (SGN-35). Binding of MMAE to tubulin disrupts the microtubule network, induces cell-cycle arrest, and results in apoptotic death of the CD30-expressing tumor cells [57]. In a phase II study, 58 patients with R/R ALCL treated with single agent brentuximab vedotin (1.8 mg/kg) showed an ORR of 86%, including a CR in 57% of patients [41]. Neutropenia (21%), thrombocytopenia (14%), and peripheral neuropathy (41%) were the most common AEs. However, resolution or improvement of peripheral neuropathy symptoms was reported in 91% of patients. The results from the five-year follow-up demonstrated that brentuximab vedotin may be a curative option for R/R ALCL patients [42]. ALCL is characterized by high CD30 expression, [55] whereas other PTCL have variable CD30 expression [58]. In a phase II study, 32 patients with MF and SS were treated with brentuximab vedotin every three weeks for a maximum of 16 doses [43]. ORR was observed in 21 patients (70%), with one CR, and seven patients (23%) showing skin improvement. Peripheral neuropathy was the most common AE occurring in 66% of patients. Additionally, a correlation between CD30 expression and response was reported in this study. Patients with a CD30 expression lower than 5% had a decreased probability of response compared to patients with a CD30 expression higher than 5%. In another prospective phase II study, 35 patients with PTCL, specifically angioimmunoblastic T cell lymphoma (AITL = 13) and PTCL-NOS (n = 22), were included [44]. The ORR was 54% in AITL and 33% in PTCL-NOS, with CR rates of 38% and 14%, respectively. Consistent with previous data, the most frequent AEs reported were neutropenia (14%) and peripheral neuropathy (9%).

In a phase I study, 39 patients with PTCL were included [45]. Six patients had ALK^pos^ ALCL, 26 patients had ALK^neg^ ALCL, and seven patients had other CD30+ PTCL. This study evaluated the activity of a sequential treatment approach (two cycles), followed by CHOP (six cycles), or a combined treatment approach of brentuximab vedotin plus CHP (CHOP without vincristine, six cycles). After sequential treatment, 11 of 13 patients achieved an objective response (CR 62%) and 23 of 26 patients treated with the combined approach achieved CR (88%). Peripheral neuropathy was experienced by 77% of patients after sequential treatment and by 69% of patients after the combined approach. The most common AEs of grade 3/4, observed in both treatment approaches, were febrile neutropenia (15–31%), neutropenia (15–23%), and anemia (15%). The five-year follow-up demonstrated a durable remission in 50% of patients treated with the combined approach [46]. Moreover, a multicenter phase III clinical trial (ECHELON-2, NCT01777152), comparing the efficacy and safety of brentuximab vedotin and CHP versus CHOP, was completed and results are expected in the near future.

A phase III, randomized, open-label, multicenter clinical trial (ALCANZA, NCT01578499) was designed to evaluate single-agent brentuximab vedotin versus a control arm of the investigator’s choice of standard therapies, methotrexate or bexarotene, in patients with CD30-expressing primary cutaneous ALCL or MF [47]. ALCANZA demonstrated an improvement in the rate of objective response, lasting at least four months (ORR4) in the brentuximab vedotin arm (ORR4 of 56%) compared to the investigator’s choice arm (ORR4 of 12%). The rate of CR was also superior in the brentuximab vedotin arm at 16%, compared to 2% in the investigator’s choice arm. The median PFS was 15.8 months in the brentuximab vedotin arm versus 3.6 months in the physician’s choice arm. Treatment-related AEs were similar between the two groups and occurred in 29% of patients. Peripheral neuropathy was reported in 67% of patients in the brentuximab vedotin group and in 6% of patients in the physician’s choice arm. At the last follow-up (median 22.9 months), improvement or resolution of peripheral neuropathy was observed in 82% of patients in the brentuximab vedotin group. 

Based on the results of the phase III ALCANZA clinical trial, in November 2017, the FDA granted regular approval to brentuximab vedotin for the treatment of adult patients with primary cutaneous ALCL or CD30-expressing MF who have received prior systemic therapy.

### 2.3. Mogamulizumab (Anti-CCR4 Monoclonal Antibody)

Mogamulizumab is a humanized monoclonal antibody directed against CC chemokine receptor 4 (CCR4), which is expressed in regulatory T cells (Treg) and T helper cells. CCR4 is also expressed in approximately 90% of patients with ATL [59] and 40% of patients with CTCL [60] and PTCL [61,62]. A phase II study of the agent was conducted in 27 patients with relapsed, aggressive CCR4-positive ATL (Table 1) [48]. ORR was observed in 50% of the 26 evaluable patients, including eight CR. The median PFS and OS were 5.2 and 13.7 months, respectively. The most commonly reported AEs were infusion reactions (89%) and skin rashes (63%). 

A phase I/II study with mogamulizumab was performed in 41 pretreated patients with CTCL [49]. The ORR in the 38 evaluable patients was 37%, 47.1% in SS (n = 17), and 28.6% in MF (n = 21). Nausea (31.0%), chills (23.8%), and infusion-related reaction (21.4%) were reported as the most common AEs in this study.

A multicenter phase II study was performed on patients with relapsed CCR4-positive PTCL (n = 29) and CTCL (n = 8) [50]. Mogamulizumab (1 mg/kg) was administered intravenously once per week for eight weeks. An ORR of 35% was observed, including five patients (14%) with CR and a median PFS of three months. The most frequent AEs of grade 3/4 were lymphocytopenia (73%), leukocytopenia (43%), and neutropenia (19%).

A phase III randomized, open-label, multinational clinical trial—MAVORIC [NCT01728805]—to compare mogamulizumab to vorinostat in previously treated CTCL, is ongoing [51]. The trial included 372 patients with MF or SS. In the first report of the clinical trial, significant improvement in ORR was found with mogamulizumab versus vorinostat in patients with both MF (21.0% vs. 7.1%, respectively) and SS (37.0% vs. 2.3%, respectively). The most common AEs in the mogamulizumab versus vorinostat included infusion related reaction (33.2% vs. 0.5%, respectively) and skin eruptions due to drug (23.9% vs. 0.5%, respectively). Mogamulizumab was first approved in 2012 in Japan for ATL and in 2014 for CTCL. In 2017, the FDA granted breakthrough therapy priority review status to mogamulizumab for the treatment of MF and SS in patients who have received at least one prior systemic therapy.

## 3. Immune Checkpoint Inhibitors

A paradigm shift in immune-oncology occurred over the past few years with the approval of monoclonal antibodies that do not target the tumor cells directly, but that enhance the anti-tumor response of the immune system by targeting immune regulatory pathways. PD-1 and its ligands are aberrantly expressed in cancer cells and in the tumor microenvironment. In T-NHL, the tumor microenvironment plays a special role and sometimes defines the tumor itself (AITL) [63]. Many T-NHL subtypes retain strong tissue tropism, gene expression profiles, and cytokine secretion patterns after transformation, which all impact the cellular composition and structure of the lymphoma. PD-L1 expression was found in different T-NHL subtypes, including PTCL (15% of cases), CTCL (27%), NKTCL (67%), ATL (25%), ALK^pos^ ALCL, and ALK^neg^ ALCL (50% and 67%, respectively) [64,65,66,67,68]. In NKTCL, an association between strong PD-L1 expression and Epstein-Barr virus infection was reported [69]. Specifically, EBV latent membrane protein 1 (LMP1) upregulation of PD-L1 through the MAPK/NF-kB pathway was reported. High PD-L1 expression and increased post-treatment serum PD-L1 levels have been proposed as biomarkers for poor prognosis in two independent studies [69,70]. However, in another study, increased OS in EBV+ NKTCL patients with high PD-L1 was observed [71]. Therefore, the prognostic value of PD-L1 in NKTCL requires further investigation. In ATL, aberrant PD-L1 expression upon 3′-untraslated region (3’-UTR) truncation and PD-L1 transcripts stabilization was reported, suggesting PD-L1 3’-UTR disruption as a potential genetic marker [67]. Transcriptional regulation of PD-L1 through STAT3 was observed in ATL and ALK^pos^ ALCL [72,73]. Furthermore, MYC and STAT3 have been identified as transcriptional regulators of PD-L1 in ALK^neg^ ALCL [68]. Aberrant expression of immune checkpoint receptors involved in T cell inhibitory and exhaustion mechanisms have been observed in PTCL and NKTCL patients [74]. Precisely, overexpression of PD-1 and CTLA-4 was found in NKTCL patients, whereas upregulation of LAG-3, TIM-3, and TIGIT was observed in PTCL patients.

The expression of inhibitory ligands on tumor cells indicates a suppression of host immunity through interaction of PD-L1 with PD-1 on activated cytotoxic T cells. However, the tumor microenvironment includes heterogeneous cell populations, including dendritic cells (DC), NK, Treg, myeloid-derived T cells, and tumor associated macrophages (TAM), which might further contribute to the suppression of host immunity [75]. PD-L1 expression on tumor infiltrating monocyte-derived cells was observed in 73% of CTCL and 39% of PTCL cases [64]. Moreover, the expression of PD-L1 on immature DC led to inhibition of T cell proliferation and induction of FoxP3+ Treg. Immunostaining of ATL patient samples showed expression of PD-L1 on infiltrating TAM. Furthermore, STAT3 has been identified as a transcriptional regulator of PD-L1 expression on TAM [76]. 

Recent findings reported the role of PD-1 as a haploinsufficient tumor suppressor in T cell lymphomagenesis [77]. In vivo genetic deletion of PD-1 led to the development of a highly aggressive lymphoma in a PTCL mouse model. Moreover, genomic alterations of PD-1 have been identified in 36% of PTCL cases. These findings suggest that immunotherapy targeting of PD-1 in certain T-NHL subtypes might cause expansion of malignant clones with oncogenic activation of the TCR pathways. Therefore, evaluation of the tumor microenvironment, PD-1 genetic deletions, and TCR oncogenic alterations prior to immune checkpoint blockade need to be considered in T-NHL.

Nivolumab and pembrolizumab are the two immune checkpoint inhibitors, both targeting PD1, in the most advanced stages of clinical development in hematological malignancies. Pembrolizumab is a humanized IgG4 monoclonal antibody that binds to the PD-1 receptor, preventing its interaction with PD-L1 and PD-L2. In T-NHL, pembrolizumab has shown clinical activity in a multicenter phase II study in 24 patients with advanced stage R/R MF and SS (Table 2) [78].

Nine patients with MF and 15 patients with SS were enrolled; 90% or greater improvement in skin disease was observed in six patients. The ORR was 38%, with one patient with SS achieving CR and eight patients (MF = 5 and SS = 3) achieving a PR. The treatment was well tolerated with a toxicity profile consistent with prior pembrolizumab studies, with the exception of immune-mediated skin flare reactions in 40% of patients with SS. Two patients experienced immune mediated treatment-related serious AEs (pneumonitis and duodenitis). A phase II trial of pembrolizumab in combination with interferon-gamma is ongoing in previously treated MF and SS patients.

One report demonstrated high response rates to pembrolizumab in R/R NK/T cell lymphomas failing L-asparaginase regimens [79]. A median of seven cycles of pembrolizumab (2 mg/kg) every three weeks were administered to seven patients. Three patients achieved CR and two patients achieved PR. The only reported AE was a grade 2 skin graft-versus-host disease in one patient with previous allogeneic HSCT. 

Nivolumab is a human IgG4 monoclonal antibody that targets the PD-1 receptor. In a phase I study, 81 patients with R/R B cell lymphoma, T cell lymphoma, and multiple myeloma were treated with nivolumab (1–3 mg/kg) every two weeks [80]. Among the 23 T-NHL patients included, 13 patients had MF, 5 had PCTL, 2 had SS, and 3 had other non-CTCL. The ORR observed were 15% and 40% in MF and PTCL, respectively, with two PR in each group and one SD in the non-CTCL patients. Drug-related AEs of any grade were reported in 74% of T-NHL patients, whereas 22% of AEs were of grade 3/4. 

In R/R ALCL, three cases were published about response to anti-PD1 therapy. In the first reported case, a 35-year old patient with ALK^neg^ ALCL treated with pembrolizumab achieved CR after allogeneic HSCT without AEs [83]. In another report, a 19-year old patient diagnosed with ALK^pos^ ALCL was refractory to chemotherapy and targeted agents (brentuximab vedotin and crizotinib) and relapsed after HSTC. After nivolumab treatment, the patient achieved a CR without graft-versus-host disease (GvHD) [84]. The third documented case described a 17-year old patient with ALK^pos^ ALCL refractory to chemotherapy and ALK inhibitors (crizotinib). Treatment with nivolumab, as a third line therapy, was followed by CR for 18 months [85].

Multiple studies of immune checkpoint blockade, as monotherapy or in combination, are ongoing in several subtypes of T-NHL (Table 3), and will provide more insight on the efficacy of these treatments.

## 4. CAR-T Cell Therapies

CD30 represents an attractive and validated target for antibody-based therapies, and engineered CAR-T cells targeting CD30 have shown potent anti-lymphoma activity in preclinical studies in various tumor models [86,87,88,89]. In a phase I study of anti-CD30 CAR-T cell therapy, 18 patients (17 with HL and one with cutaneous ALCL) were enrolled (Table 2) [81]. CAR-T cell infusion was tolerated without toxicity and seven patients achieved PR and six achieved SD. Conditioning chemotherapy was reported to enhance the engraftment of transferred T cells and improve the objective response [90,91,92,93]. Notably, the patients diagnosed with relapsed primary cutaneous ALCL achieved a three-month PR after the first CAR-T cell infusion without conditioning chemotherapy.

In another phase I study of anti-CD30 CAR-T cells, nine patients with relapsed refractory CD30+ HL and NHL (seven with HL and two with CD30+ anaplastic large cell lymphoma) were enrolled [82]. Of note, seven of these patients had either relapsed or progressive disease after treatment with brentuximab vedotin. CAR-T cell infusions were well tolerated and produced no AEs attributable to the therapy. Three patients achieved CR and three patients remained in SD. Specifically, of the two patients with ALCL, one patient with ALK^pos^ ALCL had a dramatic response after the first anti-CD30 CAR-T cell infusion, and after the fourth infusion, achieved a CR that was maintained for nine months. These studies demonstrate the tolerability, safety, and potential clinical benefit of CD30 CAR-T cell therapy. Ongoing clinical trials will further elucidate the efficacy of this approach in CD30+ malignancies (Table 4).

New strategies are under investigation in order to overcome several challenges that are still limiting the therapeutic effectiveness of CAR-T cell therapies. One potential issue is the immunogenicity of CAR, which is due to the use of scFv derived from murine antibodies. Thus, humanized or “fully human” scFv regions have been developed [94], and fully human anti-CD30 CAR-T cells are under investigation in CD30+ NHL patients (Table 4, NCT03049449). 

A major limitation of CAR-T cells in the treatment of T cell malignancies is the shared expression of target antigens between normal and malignant T cells leading to CAR-T cells fratricide or profound immunodeficiency. In preclinical models, gene editing approaches, such as CRISPR/Cas9-mediated editing and transcription activator-like effector nucleases (TALENs), have been employed to target novel antigens and increase the effectiveness of CAR-T cells [95,96]. CD7 is transmembrane protein expressed in T cell leukemia and lymphomas, and in a subset of PTCL [97,98]. CRISPR/Cas9-mediated editing has been reported to enable disruption of the CD7 gene in T cells prior to transduction with a CD7-specific CAR [99]. This modification prevents fratricide without precluding T cell expansion and antitumor activity. Furthermore, genome editing has been adapted to overcome the limitation of harvesting a sufficient number of functional T cells without contamination by malignant cells. Indeed, genome editing of the target gene in malignant T cells would result in the generation of resistant clones. Double disruption of CD7 and TCR alpha chain (TRAC) using CRISPR/Cas9 in T cells results in loss of alloreactivity and GvHD potential in vivo, thus suggesting the use of allogenic T cells as a source of CAR-T cells [100]. Moreover, suicide switch systems are under investigation to enable therapy interruption, thus preventing permanent T cell aplasia and cytotoxicity mediated by CAR-T cells. Instead of targeting a pan T cell antigen, an approach where CAR-T cells target one constant region of the TCR beta chain (TRBC) has been evaluated [101]. This approach allows the eradication of malignant cells while preserving intact a substantial proportion of the T cell compartment. Two genes, TRBC1 and TRBC2, encode the TCR beta constant region and are expressed in a mutually exclusive manner. Therefore, the normal T cell compartment contains cells expressing TRBC1 or TRBC2. The identification of TRBC1 monoclonality in different T cell malignancies has led to the development of anti-TRBC1 CAR-T cells, which recognize and kill TRBC1+ normal and malignant T cells in vitro and in a xenograft mouse model, while preserving a sufficient portion of T cells (TRBC2+). A better evaluation of adverse events, such as off-target and cytokine-mediated toxicity, following infusion of CAR-T cells, requires applications of these approaches in clinical trials. Advances in T cell ex-vivo growth, genetic engineering of other T-lineage antigens, investigation of CAR immune biology, and further optimization of CAR design will enable the preclinical evaluation of the efficacy and safety of CAR-T cells in a broad range of T-NHL. 

## 5. Conclusions

Current patient outcomes highlight the need for additional therapies and novel regimens in relapsed and refractory T-NHL. Immunotherapy with monoclonal antibodies and CAR-T cells is revolutionizing oncology, and hematological malignancies offer a particularly fertile ground to evaluate this approach. The improving understanding of interplay between malignant cells and the tumor microenvironment, as well as evasion of host immune response, will provide information regarding the dynamic nature of anti-tumor immunity and lead to optimization of immunotherapy. Evidence from preclinical data and clinical trials investigating immune checkpoint inhibitors and CAR-T cell immunotherapy in T-NHL is emerging, and was summarized in this article. Major issues such as timing and sequencing, treatment duration, CAR design, and synergistic combinatory approaches are still under investigation. Results of ongoing and future trials will lead to better awareness regarding treatment safety and efficacy.

## Figures and Tables

**Table 1:** Prospective trials of monoclonal antibodies in T cell non-Hodgkin lymphoma (T-NHL).

Agent	Combination	Phase	Lymphoma Subtypes	No. of Patients	Clinical Setting	ORR (%)	CR/PR Rate (%)	Location	Ref.
Alemtuzumab	-	II	MF; SS	22	R/R	55	32/23	Europe	[31]
	-	II	PTCL	14	R/R	36	21/14	Europe	[32]
	-	II	MF; SS	39	R/R	51	18/33	Europe	[33]
	-	II	PTCL; MF	10	R/R	60	20/40	Europe	[34]
	DHAP	II	PTCL	24	R/R	50	21/29	South Korea	[35]
	CHOP-21	II	PTCL	20	newly diagnosed	80	65/15	South Korea	[36]
	CHOP-28	II	PTCL	24	newly diagnosed	75	71/4	Europe	[37]
	CHOP-14	II	PTCL	20	newly diagnosed	90	60/50	Europe	[38]
SGN-30	-	II	C-ALCL; LyP; MF	23	R/R	70	43/26	USA	[39]
	-	II	ALCL	41	R/R	17	5/12	USA	[40]
Brentuximab vedotin	-	II	ALCL	58	R/R	86	57/27	Worldwide	[41,42]
	-	II	MF SS	32	R/R	70	3/67	USA	[43]
	-	I	PTCL; AITL	35	R/R	41	23/18	USA	[44]
	CHOP-21 (sequential)	I	ALCL	13	newly diagnosed	85	62/23	Worldwide	[45]
	CHP-21 (combination)	I	ALCL; PTCL-NOS; ATL; AITL	26	newly diagnosed	100	88/12	Worldwide	[45,46]
	-	III	pcALCL; MF	66	R/R	67	16/51	Worldwide	[47]
Mogamulizumab	-	II	ATL	27	R/R	50	30/NR	Japan	[48]
	-	I/II	MF; SS	38	R/R	37	8/29	USA	[49]
	-	II	PTCL; CTCL	37	R/R	35	14/21	Japan	[50]
	-	III	MF	105	R/R	21	NR	Europe	[51]
	-	III	SS	81	R/R	37	NR	Europe	[51]

Note: ALCL, anaplastic large cell lymphoma; AITL, angioimmunoblastic T cell lymphoma; ATL, adult T cell leukemia/lymphoma; C-ALCL, cutaneous ALCL; CHOP, cyclophosphamide, doxorubicin, vincristine and prednisone; CR, complete response; CTCL, cutaneous T cell lymphoma; DHAP, dexamethasone high-dose cytarabine cisplatin; LyP, lymphomatoid papulosis; MF, mycosis fungoides; NR, not reported; ORR, overall response rate; pcALCL, primary cutaneous ALCL; PR, partial response; PTCL-NOS, peripheral T cell lymphoma-not otherwise specified; R/R, relapsed/refractory; SS, Sézary syndrome.

**Table 2:** Prospective trials of immune checkpoint agents and anti-CD30 chimeric antigen receptor (CAR)-T cell in T-NHL.

Agent	Phase	Lymphoma Subtypes	No. of Patients	Clinical Setting	ORR%	Clinical Response n			Location	Ref.
						CR	PR	SD		
Pembrolizumab	II	SS	15	R/R	27	1	3	7	USA	[78]
		MF	9	R/R	56	-	5	2		
	II	NKTCL	7	R/R	-	3	2	-	Asia	[79]
Nivolumab	I	MF	13	R/R	15	-	2	9	USA	[80]
		PTCL	5	R/R	40	-	2	-		
		SS	3	R/R	-	-	-	-		
		non CTCL	2	R/R	-	-	-	1		
Anti-CD30 CAR-T	I	HL	17	R/R	-	-	6	6	China	[81]
		C-ALCL	1	R/R	-	-	1 (3 mo)	-		
	I	HL	7	R/R	-	2	3	-	USA	[82]
		C-ALK^neg^ ALCL	1	R/R	-	-	-	-		
		ALK^pos^ ALCL	1	R/R	-	1 (9mo)	-	-		

Note: ALCL, anaplastic large cell lymphoma; ALK^pos^ ALCL, anaplastic lymphoma kinase positive ALCL; C ALCL, cutaneous ALCL; CAR, chimeric antigen receptor; CR, complete response; CTCL, cutaneous T cell lymphoma; HL, Hodgkin lymphoma; MF, mycosis fungoides; mo, month; NKTCL, natural killer/T cell lymphoma; ORR, overall response rate; PR, partial response; PTCL, peripheral T cell lymphoma; R/R, relapsed/refractory; SD, stable disease; SS, Sézary syndrome.

**Table 3:** Open clinical trials of immune checkpoint agents in T-NHL.

Clinical Trial Identifier	Immune Checkpoint Inhibitor	Combination	Phase	Lymphoma Subtypes	Clinical Setting	No. of Patients	Location
NCT03063632	Pembrolizumab	Interferon Gamma-1b	II	MF SS	R/R	36	USA
NCT03240211		Pralatrexate and Decitabine	I	PTCL CTCL	R/R	42	Worldwide
NCT03385226		Radiotherapy	II	CTCL MF SS	R/R	46	Europe
NCT03278782		Romidepsin	I/II	PTCL CTCL	R/R	39	USA
NCT02362997		-	II	PTCL	R/R- ASCT	60	USA
NCT03021057		-	II	NKTCL	R/R	33	China
NCT03107962		-	II	NKTCL	R/R	20	China
NCT03075553	Nivolumab	-	II	PTCL	R/R	39	USA
NCT02581631		Brentuximab vedotin	I/II	PTCL CTCL	R/R	146	Worldwide
NCT02556463	Durvalumab	MEDI9197 (TLR7/8 agonist)	I	CTCL	R/R	135	Worldwide
NCT03235869		Radiotherapy	I	CTCL	untreated or R/R	19	USA
NCT03011814		Lenalidomide	I/II	PTCL CTCL	R/R	62	USA
NCT03054532		Lenalidomide	II	NKTCL	R/R	22	Singapore
NCT03161223		Pralatrexate, Romidepsin, 5-Azacitidine	I/II	PTCL	R/R	148	Worldwide
NCT03046953	Avelumab	-	II	PTCL	R/R	35	Europe
NCT03439501		-	III/IV	ENKTL	R/R	33	South Korea

Note: Information derived from www.clinicaltrials.gov database on 28 February 2018. ASCT, autologous stem cell transplantation; CTCL, cutaneous T cell lymphoma; ENKTL, extranodal natural killer/T cell lymphoma; MF, mycosis fungoides; NKTCL, natural killer/T cell lymphoma; PTCL, peripheral T cell lymphoma; R/R, relapsed/refractory; SS, Sézary syndrome.

**Table 4:** Open clinical trials of anti-CD30 CAR-T cell therapies in T-NHL.

Clinical Trial Identifier	Agent	Trial Title	Phase	Lymphoma Subtypes	Clinical Setting	No. of Patients	Location
NCT02917083	anti-CD30 CAR-T cells	Phase I study of relapsed CD30 expressing lymphomas treated with CD30 CAR-T cells (RELY-30)	I	CD30+ HL and NHL	R/R	18	Houston Methodist Hospital, Texas Children’s Hospital, Houston, Texas, United States
NCT03383965	anti-CD30 CAR-T cells	A clinical study of CD30 targeted CAR-T in treating CD30-expressing lymphomas	I	CD30+ HL and NHL	R/R	20	Weifang People’s Hospital, Weifang, Shandong, China
NCT03049449	anti-CD30 CAR-T cells	T Cells expressing a fully-human anti-CD30 chimeric antigen receptor for treating CD30-expressing lymphomas	I	CD30+ HL and NHL	R/R	76	National Institutes of Health Clinical Center, Bethesda, Maryland, United States
NCT02663297	anti-CD30 CAR-T cells	Phase I study of the administration of T lymphocytes expressing the CD30 chimeric antigen receptor (CAR) for prevention of relapse of CD30+ lymphomas after high dose therapy and autologous stem transplantation (ATLAS)	I	CD30+ HL and NHL	R/R	18	Lineberger Comprehensive Cancer Center at University of North Carolina, Chapel Hill, United States
NCT02259556	anti-CD30 CAR-T cells	CD30-directed chimeric antigen receptor T (CART30) therapy in relapsed and refractory CD30 positive lymphomas	I/II	CD30+ HL and NHL	R/R	30	Chinese PLA General Hospital, Beijing, China
NCT02690545	anti-CD30 CAR-T cells	Phase Ib/II study of the administration of T lymphocytes expressing the CD30 CAR for relapsed/refractory CD30+ Hodgkin’s Lymphoma and CD30+ Non-Hodgkin’s Lymphoma	I/II	CD30+ HL and NHL	R/R	31	Lineberger Comprehensive Cancer Center at University of North Carolina, Chapel Hill, United States
NCT02958410	anti-CD30 CAR-T cells	Clinical research of CD30-targeted CAR-T in lymphocyte malignancies	I/II	CD30+ HL and NHL	R/R	45	Southwest Hospital of Third Military Medical University, Chongqing, China

Note: Information derived from www.clinicaltrials.gov database on 28 February 2018. CAR, chimeric antigen receptor; HL, Hodgkin lymphoma; NHL, non-Hodgkin lymphoma; R/R, relapsed/refractory.
